# For Lesions of the Mucous Membrane

**Published:** 1901-04-15

**Authors:** 


### FOR LESIONS OF THE MUCOUS MEMBRANE.

We are likely to forget the value of some drugs—long in use—for the treatment of lesions of the mucous membrane, in a mad rush for the newer drugs. Many times an habitual smoker will present lesions of the tongue and cheeks which will respond to very simple treatment, as the use of two five-grain tablets of Chlorate of Potash, dissolved slowly in the mouth each day for a week. This is also a good treatment for other abrasions of the mouth and especially for syphilitic sore mouth. It is useful for extensive lacerations of the gums in treating pyorrhœa alveolaris. For thickened or tumid gums which bleed easily, dry and paint them with Compound Tincture of Iodin every other day for a week or two, and have the patient use the following formula as a spray two or three times daily:

| | | |
|---|---|---|
| ℞ | Hydronapthol, - - - | gr. xx |
| | Eucalyptol, - - - - | min. v |
| | Oil of Cloves, - - - | min. jjj |
| | Alcohol, - - - - - | ℥ ss |
| | Glycerin, - - - - | ʒ jj |
| | Distilled water, - - - | ℥ jv |

Use full strength as a spray for mouth and throat.

It is a good plan to rinse the mouth with a weak solution of Soda before using the above. If the membrane is red or slightly inflamed, use a dilute solution, 1 to 300, of Acetic Acid—common vinegar, diluted, will answer.

In cases of excoriation around the inner surfaces of the angles of the lips, use Pyoktanin, yellow. Plaster it over the rough and denuded surfaces and leave it for two or three minutes.

In simple gingivitis nothing is better than painting the entire gums with a saturated solution of Potassium

Iodide ; it does not matter if the patient should swallow a little of it. Do this daily for four or five days. Then give Soziodol internally, three grains three times each day, after meals.

For a tongue that looks badly, scrub it with dilute lemon juice, and then use Lime water or milk of Magnesia, as a mouth wash, or fill the mouth with powdered Carbonate of Magnesia. If the bowels are at fault, suggest a suitable aparient or cathartic. Dilute Ferripyrine will stop bleeding from the gums. Tincture of Matico will also do this.—Editorial in *Dental Review* for January.

